# Ezh2-mediated repression of a transcriptional pathway upstream of *Mmp9* maintains integrity of the developing vasculature

**DOI:** 10.1242/dev.112607

**Published:** 2014-12

**Authors:** Paul Delgado-Olguín, Lan T. Dang, Daniel He, Sean Thomas, Lijun Chi, Tatyana Sukonnik, Nadiya Khyzha, Marc-Werner Dobenecker, Jason E. Fish, Benoit G. Bruneau

**Affiliations:** 1 Gladstone Institute of Cardiovascular Disease, and Roddenberry Center for Stem Cell Biology and Medicine at Gladstone, San Francisco, CA 94158, USA; 2 Program in Physiology and Experimental Medicine, The Hospital for Sick Children, Toronto, Ontario M5G0A4, Canada; 3 Department of Molecular Genetics, University of Toronto, Toronto, Ontario M5S 1A8, Canada; 4 Heart and Stroke Richard Lewar Centre of Excellence, Toronto, Ontario M5G 1L7, Canada; 5 Toronto General Research Institute, University Health Network, Toronto, Ontario M5G 1L7, Canada; 6 Department of Laboratory Medicine and Pathobiology, University of Toronto, Toronto, Ontario M5S 1A1, Canada; 7 Laboratory of Lymphocyte Signaling, The Rockefeller University, New York, NY 10021, USA; 8 Department of Pediatrics, Cardiovascular Research Institute, and Institute for Regeneration Medicine, University of California San Francisco, San Francisco, CA 94158, USA

**Keywords:** Ezh2, Epigenetics, Histone methylation, Vascular development, Vascular stability, Endothelium, Extracellular matrix, Mmp9, Mouse

## Abstract

Maintenance of vascular integrity is required for embryogenesis and organ homeostasis. However, the gene expression programs that stabilize blood vessels are poorly understood. Here, we show that the histone methyltransferase Ezh2 maintains integrity of the developing vasculature by repressing a transcriptional program that activates expression of *Mmp9*. Inactivation of *Ezh2* in developing mouse endothelium caused embryonic lethality with compromised vascular integrity and increased extracellular matrix degradation. Genome-wide approaches showed that Ezh2 targets *Mmp9* and its activators *Fosl1* and *Klf5*. In addition, we uncovered *Creb3l1* as an Ezh2 target that directly activates *Mmp9* gene expression in the endothelium. Furthermore, genetic inactivation of *Mmp9* rescued vascular integrity defects in Ezh2-deficient embryos. Thus, epigenetic repression of *Creb3l1*, *Fosl1*, *Klf5* and *Mmp9* by Ezh2 in endothelial cells maintains the integrity of the developing vasculature, potentially linking this transcriptional network to diseases with compromised vascular integrity.

## INTRODUCTION

Stability of the vasculature is essential for embryonic development and tissue homeostasis. Although the establishment of vascular cell fate, differentiation, and new vessel formation has been extensively investigated, the mechanisms that stabilize the developing vasculature are less understood. Endothelial extracellular matrix (ECM) homeostasis is key for vascular stability during development (Ingram et al., 2013), as the ECM provides a scaffold that supports the organization of endothelial cells into blood vessels (Davis and Senger, 2005), and its degradation by increased activity of matrix metalloproteinases (MMPs) compromises embryonic development (Chang et al., 2006) and cardiovascular function (Spinale et al., 2013). Mmp9 is an important regulator of ECM homeostasis in development and disease. Increased activity of Mmp9 compromises vascular integrity in cardiovascular pathologies including aortic aneurysm (Duellman et al., 2012; Longo et al., 2002), and can promote rupture of atherosclerotic plaques (Gough et al., 2006). Thus, *MMP9* expression must be kept in check to maintain vascular integrity.

Gene expression programs are stabilized by repressive histone methylation (Black et al., 2012), which is required for long-term organ homeostasis (Delgado-Olguin et al., 2012). The polycomb repressive complex 2 (PRC2), which tri-methylates lysine 27 of histone H3 (H3K27me3) through Ezh2, regulates angiogenesis and has been indirectly associated with *MMP9* expression. Ezh2 promotes angiogenesis in ovarian carcinoma (Lu et al., 2007, 2010) and glioblastoma cells (Smits et al., 2011, 2010). In human umbilical vein endothelial cells (HUVECs), Ezh2 also promotes angiogenesis by regulating cell adhesion and communication (Dreger et al., 2012). By contrast, Ezh2 inhibits endothelial differentiation and angiogenesis *in vitro* in Ewing tumor cells (Richter et al., 2009). In addition, Ezh2-mediated repression of tissue inhibitors of metalloproteinases (TIMPs) indirectly promotes Mmp9 activity in prostate cancer cells (Shin and Kim, 2012). *MMP9* is epigenetically regulated by DNA methylation and histone acetylation in cancer cells (Labrie and St-Pierre, 2013). However, whether Ezh2 controls the expression of *MMP9* or its transcriptional activators in developing endothelium, or whether Ezh2 has a function in vascular development and maintenance are unknown.

Transcriptional activators of *MMP9* in non-endothelial cells include the leucine zipper protein FOS-like antigen 1 (Fosl1) (Kent et al., 2011), the zinc-finger protein Kruppel-like factor 5 (Klf5) (Shinoda et al., 2008) and cAMP response element-binding protein (Creb). Fosl1 activates *MMP9* expression in trophoblasts, Klf5 in cartilage and Creb in mesothelial cells (Shukla et al., 2009). In addition, Klf5 is linked to vascular inflammation (Lu et al., 2013), aortic aneurysm and heart failure (Haldar et al., 2010), and Creb enhances inflammation in a model of atherosclerosis (Kotla et al., 2013), suggesting functions in vascular maintenance. However, whether Fosl1, Klf5 or Creb-like proteins are regulated in endothelial cells, or whether they are involved in the maintenance of the developing vasculature, is unknown. Uncovering key regulators of *MMP9* in endothelial cells could provide insight into vasculature development and maintenance, and into the mechanisms of cardiovascular disease.

## RESULTS

### Ezh2 is required for vascular integrity

*Ezh2* was inactivated in developing endothelial progenitor cells via *Tie2::cre*-mediated homologous recombination. Efficient *Ezh2* inactivation was revealed by significantly decreased H3K27me3 immunofluorescence signal in Pecam-expressing cells of E10.5 embryos (supplementary material Fig. S1). High-throughput sequencing of RNA (RNA-seq) revealed higher expression of *Ezh2* than *Ezh1* (22.48 versus 2.92 fragments per kilobase of exon per million fragments mapped, or FPKM) in sorted endothelial cells, and the expression of *Ezh1* did not change upon *Ezh2* deletion (*P*>0.5), indicating that Ezh2 is the major H3K27me3 methyltransferase in developing endothelial cells. Overall normal vascular patterning in *Ezh2* mutants, as shown by whole-mount immunostaining of Pecam on E10.5 embryos (supplementary material Fig. S2), suggests that *Ezh2* is not crucial for endothelial cell differentiation in the developing vasculature. However, homozygous mutant embryos died between E13.5 and E14.5 (supplementary material Table S1), indicating an essential function for endothelial *Ezh2* in the later stages of vascular development.

**Fig. 1.**
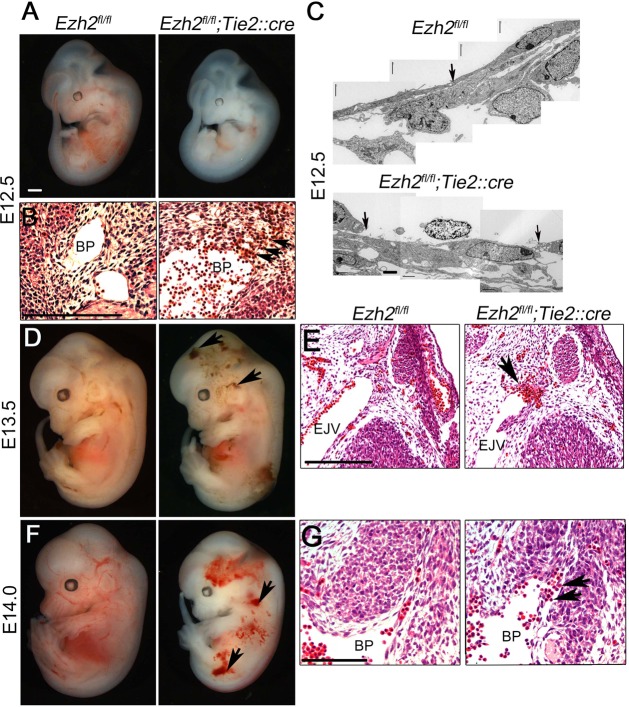
**Ezh2 is essential for vascular integrity.** (A) Control (*Ezh2^fl/fl^*) and mutant (*Ezh2^fl/fl^;Tie2::cre*) embryos at embryonic day 12.5 (E12.5). Mutant embryos appear anemic when compared with controls. (B) Higher magnification section of the brachial plexus (BP) showing extravasated blood cells (arrows). (C) Transmission electron micrograph showing a disrupted cellular junction (arrows) in mutant (*Ezh2^fl/fl^;Tie2::cre*), but not control (*Ezh2^fl/fl^*), embryos. (D,F) Whole-mount control and mutant embryos at E13.5 and 14.0, respectively, showing hemorrhages (arrows). (E,G) Higher magnification sections of the external jugular vein (EJV) and brachial plexus (BP) of E13.5 and E14.0 embryos, respectively, showing extravasated blood cells (arrows). Scale bars: 200 μm in A,B,D-G; 1 μm in C.

Consistent with the involvement of *Ezh2* in erythropoiesis in the developing liver (Mochizuki-Kashio et al., 2011), *Ezh2* mutant embryos appeared anemic (Fig. 1; supplementary material Fig. S3A). In addition, E11.0 mutant embryos had abnormal endocardial arrangement, with a gap present between the endocardium and myocardium (supplementary material Fig. S3B). At E12.5, embryos appeared anemic and had internal hemorrhaging, with extravasated red blood cells in the mesenchyme surrounding the brachial plexus (Fig. 1A,B). Furthermore, electron microscopy revealed gaps in the endothelium lining the brachial plexus (Fig. 1C). At E13.5, 83% of mutant embryos had superficial hemorrhages (Fig. 1D) and a thinner ventricular wall (supplementary material Fig. S3C,D). In addition, internal hemorrhaging was present, with extravasated red blood cells surrounding the external jugular vein (Fig. 1D,E). E14 mutant embryos died of severe superficial hemorrhaging and had extravasated red blood cells surrounding the brachial plexus (Fig. 1F,G). Immunostaining for phosphorylated histone H3 and activated caspase 3 in Pecam-expressing endothelial cells was comparable between control and mutant embryos (supplementary material Fig. S4), suggesting that Ezh2 deficiency does not affect endothelial cell proliferation or induce apoptosis. Thus, Ezh2 is required for the maintenance of vascular integrity during development.

### Ezh2 represses regulators of ECM remodeling in developing endothelium

**Fig. 2.**
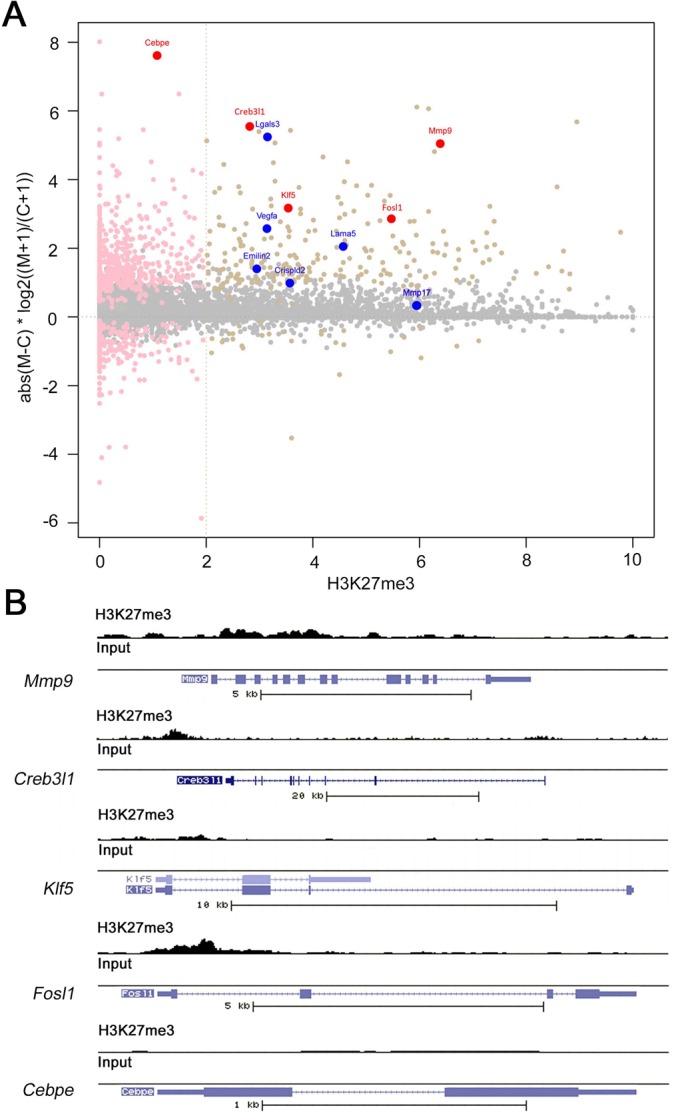
**Ezh2 represses regulators of ECM remodeling.** (A) Relationship between differential expression and H3K27me3. Pink dots indicate differential expression at *P*<0.01 and beige dots indicate genes that were both differentially expressed and marked with K27me3 at a threshold of 2. Blue dots indicate genes involved in ECM remodeling, as identified by DAVID analysis; red dots indicate *Mmp9* and its activators. (B) Mapping of H3K27me3-marked chromatin reads over the genomic region of *Mmp9*, *Creb3l1*, *Klf5*, *Fosl1* and *Cebpe*. Solid boxes represent exons. Arrows indicate direction of transcription. *Mmp9*, *Creb3l1*, *Klf5* and *Fosl1* were enriched in H3K27me3.

As a first approach to uncover the mechanistic involvement of Ezh2 in vascular integrity, we analyzed the global gene expression pattern of endothelial cells sorted from E10.5 mouse embryos. At this developmental time-point, the vascular integrity defects are not yet apparent in Ezh2 endothelial-specific knockouts. Endothelial cells were genetically labeled by crossing *ROSA26^mT/mG^* transgenic mice, which have a reporter driving constitutive expression of membrane Tomato and a Cre-inducible membrane *GFP* (Muzumdar et al., 2007), with *Tie2::cre* mice. *Ezh2^fl/fl^* females were crossed with *Ezh2^fl/+^;Tie2::cre; ROSA26^mT/mG^* males to obtain control *Ezh2^fl/+^;Tie2::cre;ROSA26^mT/mG^* and mutant *Ezh2^fl/fl^;Tie2::cre;ROSA26^mT/mG^* embryos. To exclude the effect of *Ezh2* deletion on erythropoiesis, the liver primordium was dissected out (supplementary material Fig. S5A) before disassociation of embryos. The GFP-positive cell population sorted from *Ezh2* mutants (supplementary material Fig. S5B) efficiently deleted *Ezh2* and had decreased levels of *Ezh2* mRNA, as shown by RT-PCR and real-time quantitative PCR (qPCR), respectively (supplementary material Fig. S5C,D). In addition, the endothelial markers *Flk1* and *Pecam* were enriched in the sorted cell population when compared with whole embryos (supplementary material Fig. S5E), indicating that the isolated cell population is enriched in endothelial cells. RNA-seq analysis revealed that Ezh2-deficient endothelial cells misregulated 1084 genes, of which 858 were upregulated and 226 downregulated (Fig. 2A; supplementary material Table S2). Functional annotation of the upregulated genes using DAVID (Huang et al., 2009a,b) revealed enriched gene ontology categories related to cell adhesion and ECM remodeling (supplementary material Fig. S6), suggesting potentially altered functions leading to instability of the *Ezh2*-deficient endothelial cell layer. Indeed, *Mmp9*, an important mediator of ECM degradation in normal physiology and diseases associated with compromised vascular integrity (Duellman et al., 2012; Gough et al., 2006; Longo et al., 2002), was upregulated. Furthermore, *Fosl1* and *Klf5*, known *Mmp9* activators in non-endothelial contexts (Kent et al., 2011; Shinoda et al., 2008; Shukla et al., 2009), and the transcription factor cAMP-responsive element binding protein 3-like 1, or *Creb3l1*, were also upregulated (Fig. 2A; supplementary material Table S2).

To identify direct targets of Ezh2 function, we immunoprecipitated H3K27me3-enriched chromatin followed by high-throughput sequencing (ChIP-seq). Chromatin was immunoprecipitated from endothelial GFP-positive cells sorted from E10.5 *Ezh2^+/+^;Tie2::cre;ROSA26^mT/mG^* embryos. ChIP-seq identified 3575 genes marked with H3K27me3. Such genes include previously identified targets of PRC2 in other contexts such as the pluripotency regulator *Sox2*, Hox genes and the homeodomain transcription factor *Six1* (Boyer et al., 2006; Delgado-Olguin et al., 2012) (supplementary material Fig. S7 and Table S2). Overall, H3K27me3-marked genes tended to be expressed at lower levels in both control and *Ezh2*-deficient cells when compared with genes lacking H3K27me3 (supplementary material Fig. S8A). In addition, the analysis revealed that genes marked by H3K27me3 were not predisposed towards differential expression in Ezh2-deficient endothelial cells (supplementary material Fig. S8B). However, a subset of 231 genes marked with H3K27me3 was significantly upregulated, and only 29 genes were downregulated (Fig. 2A, supplementary material Table S2). H3K27me3-marked upregulated genes include those involved in ECM remodeling identified by functional annotation (Fig. 2A,B). Furthermore, *Fosl1*, *Klf5* and *Creb3l1*, which were strongly activated in *Ezh2*-deficient endothelial cells, were identified as direct Ezh2 targets (Fig. 2B; supplementary material Table S2). However, CCAAT/enhancer-binding protein ε (*Cebpe*) was not marked by H3K27me3. Thus, Ezh2 appears to repress a transcriptional pathway that promotes ECM degradation and disrupts integrity of the developing vasculature.

### Ezh2-deficient endothelium has increased gelatinase/collagenase activity and upregulates *Mmp9* activators

**Fig. 3.**
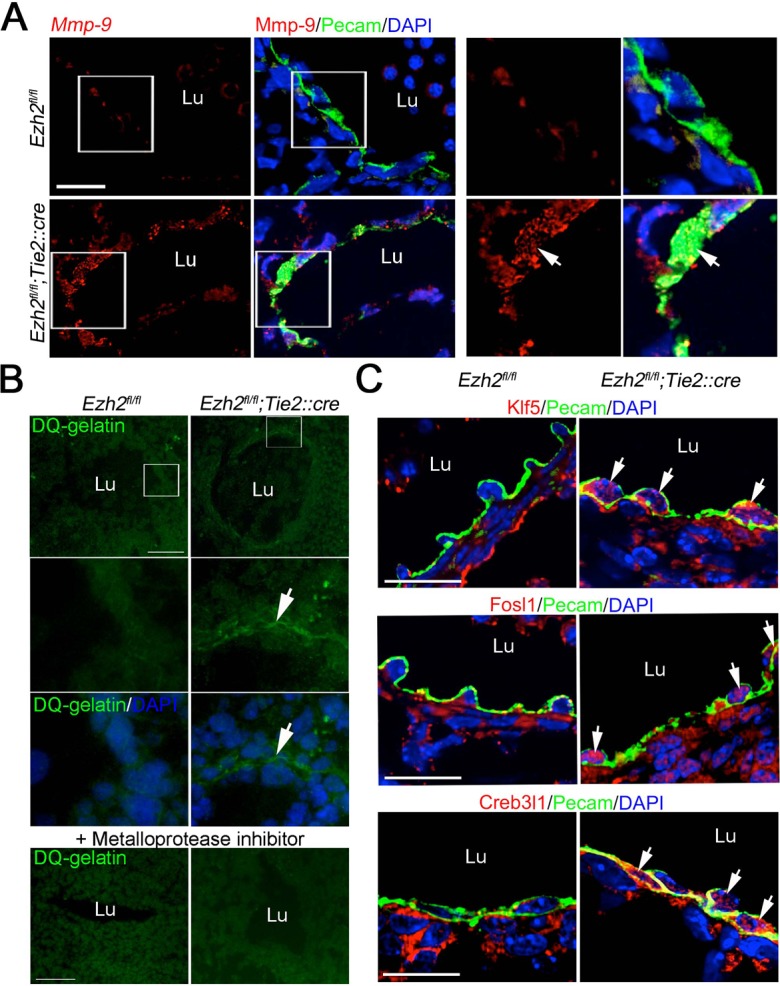
**Ezh2-deficient endothelium has increased gelatinase/collagenase activity and upregulates *Mmp9* activators.** (A) Immunofluorescence for Mmp9 on E11.0 control (*Ezh2^fl/fl^*) and mutant (*Ezh2^fl/fl^;Tie2::cre*) embryo sections. Pecam labels endothelial cells. Panels on the right are higher magnifications of the boxed areas on the left. Nuclei were stained with DAPI. Scale bar: 20 μm. (B) *In situ* zymography on sections from control and mutant E11.0 embryos. Sections were overlaid with DQ-gelatin, which revealed gelatinase/collagenase activity (arrows) surrounding the cells lining the vasculature lumen (Lu). Addition of a metalloprotease inhibitor blocked DQ-gelatin fluorescence. Nuclei were stained with DAPI. Scale bar: 50 μm. (C) Immunofluorescence of Mmp9 Klf5, Fosl1 and Creb3l1 on E11.5 mouse embryo sections. Scale bars: 20 μm.

Upregulation of *Mmp9* and its transcriptional activators suggests that Ezh2-deficient endothelium may have increased extracellular matrix degradation. To test this hypothesis, we first assessed the distribution of Mmp9 in wild-type and *Ezh2*-deficient endothelium in sections of E11.0 embryos by immunofluorescence. Mmp9 was detected in the cytoplasm of endothelial cells of mutant, but not control, embryos (Fig. 3A). ECM degradation was assessed by *in situ* zymography, which detects gelatinase/collagenase activity, on sections of E11.0 embryos. Activity was detected in the *Ezh2*-deficient endothelial cell layer that lines the jugular vein, but not the normal endothelium (Fig. 3B). Preincubating the tissue sections with a metalloprotease inhibitor blocked gelatinase/collagenase activity in *Ezh2*-deficient endothelium (Fig. 3B). These results suggest increased ECM remodeling in Ezh2-deficient endothelium.

To confirm that Klf5, Fosl1 and Creb3l1 are induced at the protein level in Ezh2-deficient endothelium, we assessed their expression by immunofluorescence on sections of E11.0 embryos. Although these proteins were present at very low levels in control endothelial cells, increased signal was detected in *Ezh2*-deficient endothelial cells (Fig. 3C). Increased expression of *Mmp9*, *Klf5*, *Fosl1* and *Creb3l1* mRNAs in Ezh2-deficient endothelium was also observed by *in situ* hybridization (supplementary material Fig. S9). It is possible that *Klf5*, *Fosl1* and *Creb3l1* activate *Mmp9* gene expression when de-repressed in *Ezh2*-deficient endothelial cells.

### Creb3l1 induces endogenous expression of *Mmp9* in endothelial cells

**Fig. 4.**
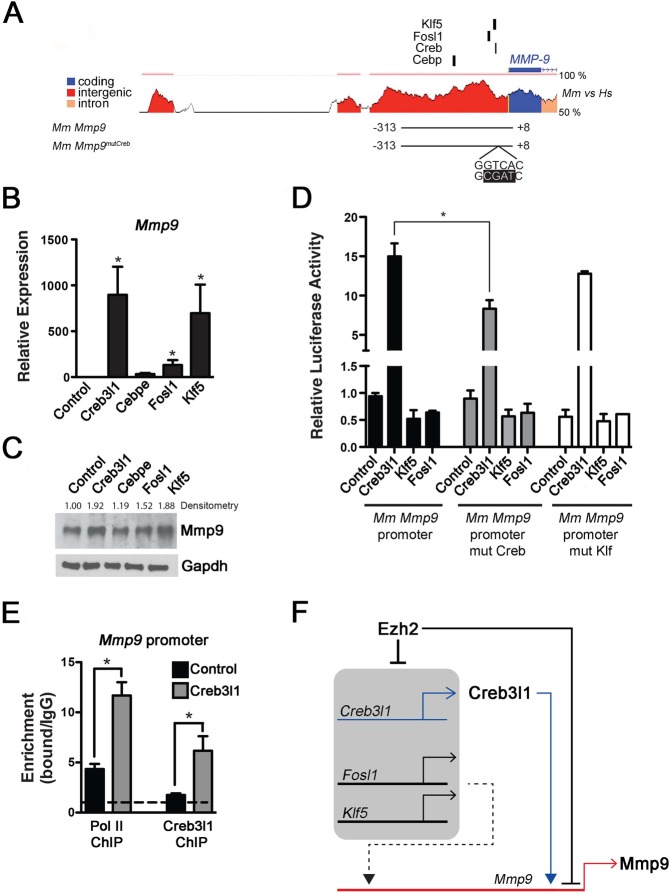
**Creb3l1, Klf5 and Fosl1 activate *Mmp9* expression in endothelial cells.** (A) ECR browser conservation plots for the 5′ end of the *Mmp9* promoter, showing percentage conservation between mouse and human. Exon 1 is indicated in blue, intron 1 in pink and the 5′ non-coding sequences in red. Black vertical lines indicate conserved binding motifs. The line beneath the plot corresponds to the mouse Mmp9 proximal promoter fragment used in luciferase reporter assays. (B) Overexpression of *Creb3l1*, *Fosl1* and *Klf5* in bovine aortic endothelial cells significantly increases expression of *Mmp9* mRNA. Error bars indicate s.e.m. (C) Western blot depicting relative increases in Mmp9 protein levels with *Creb3l1*, *Cebpe*, *Fosl1* or *Klf5* overexpression. Densitometry values are indicated, normalized to Gapdh. (D) Overexpression of *Creb3l1*, unlike *Fosl1* or *Klf5*, can induce the mouse *Mmp9* proximal promoter in a luciferase reporter assay. Mutation of the CREB-binding site significantly reduced *Mmp9* proximal promoter activity in the presence of Creb3l1. Relative luciferase assay shown is an average of triplicates of one representative experiment. (E) Overexpression of Creb3l1 increased RNA polymerase II and Creb3l1 occupancy at the *Mmp9* proximal promoter. Shown is an average of qPCR triplicates of one representative experiment. Error bars indicate s.e.m. (F) Working model of Ezh2-mediated regulation of Mmp9 expression. *Ezh2* deficiency derepresses the transcriptional activators *Creb3l1*, *Fosl1* and *Klf5*, leading to the upregulation of *Mmp9*. Whereas Creb3l1 can activate the proximal *Mmp9* promoter, Fosl1 and Klf5 may regulate more distal enhancers of the *Mmp9* gene locus or may act in an indirect manner to induce *Mmp9*. *P>0.05.

To understand the basis for transcriptional activation of *Mmp9* in Ezh2-deficient endothelial cells, we analyzed the *Mmp9* promoter looking for conserved transcription factor-binding motifs. rVista (Loots and Ovcharenko, 2004) identified conserved SP1- and AP1-binding motifs, which are recognized by Klf5 and Fosl1, respectively, in mouse and human. In addition, binding motifs for CREB and CEBP were also conserved (Fig. 4A). *Creb3l1* and *Cebpe* were among the most significantly upregulated genes in Ezh2-deficient endothelial cells, and *Fosl1* and *Klf5* were also highly upregulated (Fig. 2A). Therefore, we addressed the ability of Creb3l1, Fosl1, Klf5 and Cebpe to activate *Mmp9* gene expression in endothelial cells. cDNAs encoding these transcription factors were overexpressed in bovine aortic endothelial cells (BAECs), in which we measured endogenous *Mmp9* by qPCR and protein levels by western blot. The transfected factors were highly expressed, as confirmed by qPCR (data not shown). *Cebpe*, which is not targeted by Ezh2 (Fig. 2), did not induce *Mmp9* expression. By contrast, *Creb3l1*, *Fosl1* and *Klf5*, which are direct Ezh2 targets (Fig. 2), robustly induced *Mmp9* expression (Fig. 4B). Increased Mmp9 protein levels were also observed (Fig. 4C).

### Creb3l1 directly activates the *Mmp9* promoter in endothelial cells

To test the capacity of Creb3l1, Fosl1 and Klf5 to directly activate the expression of *Mmp9* directly we analyzed the induction of luciferase reporters driven by the mouse and human *Mmp9* and *MMP9* proximal promoters, which include conserved Creb, Fosl and Klf-binding motifs (Fig. 4A; supplementary material Fig. S10). Creb3l1, but not Klf5 or Fosl1, activated both the human and mouse proximal promoters in BAECs (Fig. 4D; supplementary material Fig. S10). It is possible that functional Fosl1- and Klf5-responsive elements are located distal to the proximal promoter. Mutation of the Creb- but not the Klf-binding motif in the human and mouse *Mmp9* and *MMP9* promoter significantly decreased activity (Fig. 4D; supplementary material Fig. S10). Furthermore, chromatin immunoprecipitation revealed interaction of Creb3l1 concomitant with increased PolII recruitment on the *Mmp9* promoter in BAECs transfected with *Creb3l1* cDNA (Fig. 4E). Thus, Ezh2 represses *Mmp9* as well as *Creb3l1*, which directly activates *Mmp9* promoter activity in endothelial cells via its conserved binding site (Fig. 4F).

### De-repression of *Mmp9* causes vasculature instability in Ezh2-deficient endothelial cells

**Fig. 5.**
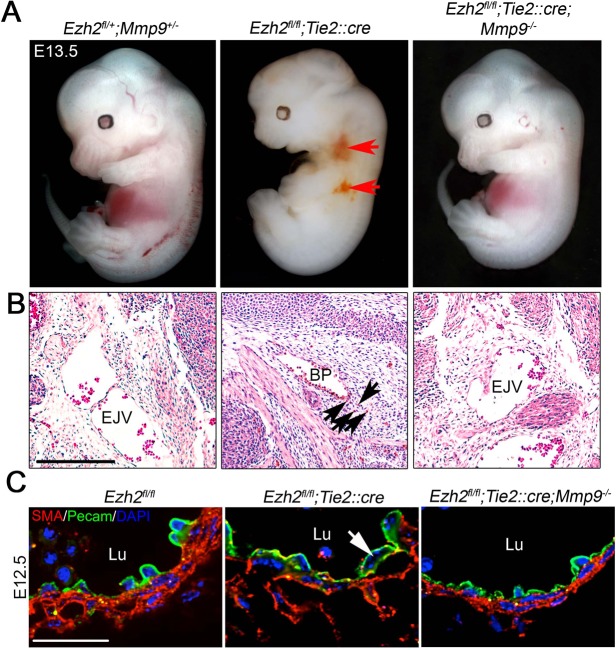
**Genetic inactivation of *Mmp9* prevents vascular instability in Ezh2-deficient embryos.** (A) E13.5 whole embryos showing hemorrhages (arrows) in a *Ezh2* mutant (*Ezh2^fl/fl^;Tie2::cre*), but not in control (*Ezh2^fll+^;Mmp9^+/−^*) or in *Ezh2* and *Mmp9* double mutants (*Ezh2^fl/fl^;Tie2::cre;Mmp9^−/−^*). (B) Hematoxylin and Eosin-stained sections showing extravasated blood cells (arrows) in a *Ezh2* mutant, but not in a control embryo or in a *Ezh2* and *Mmp9* double mutant. Scale bar: 200 μm. (C) Immunofluorescence of smooth muscle actin (SMA) and Pecam on sections of a control (*Ezh2^fl/fl^*), a *Ezh2* mutant (*Ezh2^fl/fl^;Tie2::cre*) and a *Ezh2* and *Mmp9* double mutant embryo. The arrow indicates an endothelial cell detached from the extracellular matrix. EJV, external jugular vein; BP, brachial plexus; Lu, lumen. Scale bar: 20 μm.

To test the involvement of de-repression of *Mmp9* in vascular instability caused by *Ezh2* deficiency, we genetically inactivated *Mmp9* in *Ezh2^fl/fl^;Tie2::cre* embryos by crossing *Ezh2^fl/+^;Tie2::cre;Mmp9^−/+^* males with *Ezh2^fl/fl^;Mmp9^−/−^* females. These crosses produced 19% live *Ezh2^fl/fl^;Tie2::cre;Mmp9^−/+^* embryos (as determined by presence of a heartbeat) at E13.5, which is closer to the 25% expected, than the 8% live *Ezh2^fl/fl^;Tie2::cre* embryos recovered at the same stage (supplementary material Table S1). Although 7%, of the expected 12.5% of live *Ezh2^fl/fl^;Tie2::cre;Mmp9^−/−^* embryos were recovered (supplementary material Table S3), none of these *Ezh2^fl/fl^;Tie2::cre;Mmp9^−/−^* embryos presented superficial or internal hemorrhages (Fig. 5A,B; supplementary material Table S3; *P*<0.001 by Fisher's exact test). In addition, although 83% of *Ezh2^fl/fl^;Tie2::cre* embryos were hemorrhagic, only 37% of *Ezh2^fl/fl^;Tie2::cre;Mmp9^+/−^* embryos had hemorrhages (supplementary material Table S3; *P*<0.001 by Fisher's exact test). Furthermore, the disorganization of the smooth muscle cell layer and endothelial cell detachment observed in *Ezh2^fl/fl^;Tie2::cre* embryos at E12.5 were rescued in *Ezh2^fl/fl^;Tie2::cre;Mmp9^−/−^* embryos (Fig. 5C). Altogether, our results suggest that Ezh2 represses the expression of *Mmp9* and its transcriptional activators in developing endothelial cells to limit ECM remodeling, thus promoting vascular stability during embryogenesis.

## DISCUSSION

Regulation of endothelial ECM remodeling during development is essential for embryogenesis (Lu et al., 2011), but the transcriptional mechanisms involved are poorly understood. We found that Ezh2 stabilizes the developing vasculature by repressing a transcriptional pathway that activates *Mmp9* (Fig. 4F). Other chromatin modifiers such as the histone deacetylase HDAC7 (Chang et al., 2006), the ATP-dependent chromatin remodeler BRG1 (Davis et al., 2013) and the chromodomain-helicase-DNA-binding protein 4, or CHD4, which is an ATPase of the nucleosome-remodeling and histone deacetylase (NuRD) chromatin-remodeling complex (Ingram et al., 2013), function in ECM remodeling and are required for development. Thus, epigenetic control of endothelial gene expression programs at multiple levels is required for ECM homeostasis and embryogenesis.

PRC2 targets numerous transcription factors that regulate developmental transitions in embryonic stem cells, and stably represses specific transcriptional programs that allow cell differentiation (Boyer et al., 2006; Lee et al., 2006) and maintenance (Delgado-Olguin et al., 2012). De-repression of key Ezh2 targets with the potential to activate gene regulatory networks destabilizes gene expression programs and could predispose to disease (Delgado-Olguin et al., 2012). Thus, identification of key Ezh2 targets could help to uncover regulators of vascular stability in human disease. Here, we uncovered *Creb3l1*, *Klf5* and *Fosl1* as Ezh2 targets able to activate a transcriptional program upstream of *Mmp9* (Fig. 4G), and thus likely to contribute to vascular instability. We also identified *Mmp9* as a direct target of Ezh2. The recent finding of Creb enhancing inflammation in a model of atherosclerosis (Kotla et al., 2013), coupled to the contribution of Mmp9 in atherosclerotic lesion rupture (Gough et al., 2006) and development of aortic aneurysm (Duellman et al., 2012; Longo et al., 2002), suggest that Creb-like proteins might have a function in Mmp9-related vascular disease. Indeed, both *CREB3L1* and *MMP9* are up-regulated in intracranial aneurysm (Li et al., 2009), which causes hemorrhage (Weir, 2002) and is associated with MMP-mediated destabilization of the arterial wall (Ishibashi et al., 2012). Future studies will be required to establish a causative function for Creb3l1 in vascular integrity disorders.

*Ezh2* is a known positive regulator of tumor angiogenesis. For example, Ezh2 in endothelial cells promotes vessel growth in several tumor models (Dreger et al., 2012; Lu et al., 2007, 2010; Smits et al., 2011, 2010). However, our results and those of others (Mochizuki-Kashio et al., 2011) indicate that Ezh2 does not have a central function in controlling angiogenesis during embryonic development. Instead, we found that Ezh2 maintains integrity of the developing vasculature. It is likely that gene regulatory networks controlled by Ezh2 in tumor endothelial cells differ from those in embryonic endothelial cells during the formation of blood vessels. Indeed, tumor endothelial cells have been shown to have a highly unique gene expression pattern compared with other endothelial cells (St Croix et al., 2000).

A role for Ezh2 in repressing *Mmp9* could have implications in the development of therapeutic strategies for cancer. *MMP9* is dramatically upregulated in cancer and various inflammatory conditions, and has been proposed as a potential therapeutic target (St-Pierre et al., 2004). Therefore, identifying key regulators of *MMP9* expression could provide opportunities for novel therapies. *EZH2* overexpression in various cancers correlates with tumor aggression and can serve as a prognosis indicator (Chang and Hung, 2012). Inactivation of *EZH2* inhibits metastasis, tumor angiogenesis and growth (Chang et al., 2006), and small molecule-mediated inhibition of the enzymatic function of Ezh2 may allow pharmacological treatment of cancer (Crea et al., 2012). However, as vascular instability facilitates metastasis (Nguyen et al., 2009), it is possible that Ezh2 inhibition could promote metastasis in some types of cancer. In addition, the function of *Ezh2* in cardiac maintenance (Delgado-Olguin et al., 2012) raises issues regarding the potential secondary effects of inhibiting Ezh2. Therefore, experimental therapies targeting Ezh2 should address potential secondary effects on cardiovascular maintenance.

## MATERIALS AND METHODS

### Mice

The following mouse strains were used: *Ezh2^fl/fl^* (Su et al., 2003), *Tie2::cre* (Proctor et al., 2005), *ROSA26^mT/mG^* (Muzumdar et al., 2007) and *Mmp9^−/−^* (Coussens et al., 2000). All animal experiments followed guidelines of the University of California, San Francisco Institutional Animal Care and Use Committee, and were approved by the Toronto Centre for Phenogenomics Animal Care Committee. *Ezh2^fl/fl^* and *Tie2::cre* lines were backcrossed with *Mmp9^−/−^* mutants to mix the genetic backgrounds. Embryos were dissected in PBS and fixed in 4% PFA for 2 h at 4°C, dehydrated in an ethanol series and stored at −20° until processing. Embryos were rehydrated by reversing the ethanol series and then processed for histological analysis by Haematoxylin and Eosin staining.

### RNA-seq

RNA was isolated from GFP-positive cells sorted from control *Ezh2^fl/+^;Tie2::cre;Rosa^mT/mG^* and mutant *Ezh2^fl/fl^;Tie2::cre;Rosa^mT/mG^* embryos. Cells sorted from individual embryos were used to prepare RNA-seq libraries. RNA-seq libraries were prepared using the Ovation RNA-seq System (NuGen) as recommended by the manufacturer. Differential expression was ranked by calculating the ‘diffrat’ or absolute difference times the ratio: abs(a−b)×log2((a+1)/(b+1)). Differential expression significance was calculated using rank expectation, at multiple-testing-adjusted *P* value of *P*≤0.01 (Thomas et al., 2011).

### ChIP-seq

ChIP was performed as previously described (O'Geen et al., 2011). Three million GFP-positive cells sorted from E10.5 *Tie2::cre;Rosa^mT/mG^* embryos were used. For data analysis, tag density was calculated within 2 kb of each transcription start site and the values were converted to log scale. For cross-referencing of RNA-seq and ChIP-seq datasets, FPKM values for each RNAseq dataset were calculated for each gene and then log normalized to a 0 to 10 point scale, with 10 representing the smallest value that captured 95% of the data. For each transcription start site, the number of H3K27me3 ChIP-seq tags within 2 kb of the transcription start site was counted and log normalized to a similar 10-point scale. Transcripts with values of 2/10 or less were categorized as having ‘low’ H3K27me3 signal, and genes with greater than 2/10 were categorized as having ‘high’ H3K27me3 signal at the TSS.

### Plasmids

Expression vectors for *Creb3l1*, *Klf5*, *Fosl* and *Cebpe* were from Open Biosystems. Promoter fragments were PCR amplified using KOD DNA Polymerase (Millipore) and cloned into the *Xho*I site of pGL3-Basic (Promega). Mutations in putative binding sites for Creb and Klf5 of the human and mouse *MMP9* and *Mmp9* promoter fragments were introduced using the Q5 Site-Directed Mutagenesis Kit (New England Biolabs). Primers are in supplementary material Table S4.

### Gene expression analysis

RNA was isolated from GFP-positive cells sorted from control *Ezh2^fl/+^;Tie2::cre;Rosa^mT/mG^* and mutant *Ezh2^fl/fl^;Tie2::cre;Rosa^mT/mG^* embryos, or from whole control embryos, using Trizol LS Reagent (Invitrogen) and treated with DNaseI. Isolation of RNA from cultured bovine aortic endothelial cells (BAECs) was performed similarly. cDNA was synthesized using SuperScript III First Strand Synthesis Kit (Invitrogen). cDNA (10 pg) were used for quantitative real-time PCR amplification using TaqMan probes or SYBR Green chemistry. The following TaqMan probes were used: *Ezh2* (Mm00468464_m1), *Pecam1* (Mm01242584_m1) and *Flk1* (Mm01222421_m1). Primers used for amplification with SYBR Green are in supplementary material Table S4.

### Transmission electron microscopy

Embryos were fixed in 0.1 M sodium cacodylate buffer (pH 7.4) with 2% glutaraldehyde and 1% paraformaldehyde, and post-fixed in the same buffer with 2% osmium tetroxide, then stained with 2% aqueous uranyl acetate, dehydrated in acetone, infiltrated and embedded in LX-112 resin (Ladd Research Industries). Samples were ultrathin sectioned on a Reichert Ultracut S ultramicrotome and counter stained with 0.8% lead citrate. Grids were examined on a JEOL JEM-1230 transmission electron microscope (JEOL USA) and photographed with the Gatan Ultrascan 1000 digital camera (Gatan).

### Immunofluorescence

Sections (4 μm) on glass slides were fixed with 4% PFA for 10 min, washed three time for 5 min each, blocked with 5% goat serum in PBS and incubated with primary antibodies in PBS with goat serum overnight at 4°C. After three washes with PBS, sections were incubated with secondary antibodies (Alexafluor) for 2 h at room temperature, washed and mounted with ProLong Gold Antifade Reagent (Life Technologies). Antibodies and dilutions used were: Mmp9 (C20, Santa Cruz Biotechnology, 1/100), Creb3l1 (Abcam, ab33051, 1/100), Klf5 (Abgent, ABGAP7342B, 1/100), Fosl1 (Fra1 C12, Santa Cruz Biotechnology, 1/100), phospho histone H3 (Abcam, AB5176, 1/200), activated caspase 3 (Sigma, C8487, 1/200), smooth muscle actin (Clone 1A4, A2547, Sigma, 1/200), and CD34 (Abcam, ab8158, 1/200).

### *In situ* zymography

ECM degradation was visualized using the EnzChek Gelatinase/Collagenase Assay Kit (Life Technologies). Cryosections (8 μm) obtained from fresh non-fixed E11.5 embryos were allowed to dry for 10 min, were washed with PB2 for 5 min and were covered with a solution of 1% low melting point agarose melted in PBS containing 50 μg/ml of DQ gelatin and 1 μg/ml of DAPI. Slides were coverslipped and incubated at 4°C for 5 min and then at 37°C for 6 h. Sections were imaged immediately after incubation.

### Western blot

BAEC cells were lysed in 1× RIPA buffer (50 mM Tris HCl, 150 mM NaCl, 1.0% NP-40, 0.5% sodium deoxycholate, 1.0 mM EDTA and 0.1% SDS) and diluted in Laemmli loading buffer [63 mM Tris-HCl (pH 6.8), 0.1% 2-mercaptoethanol, 0.0005% bromophenol blue, 10% glycerol and 2% SDS]. Proteins were resolved in 4-12% acrylamide gels and transferred to PVDF membranes, which were blocked with 5% skimmed milk and 0.05% Tween-20 in TBS [50 mM Tris-Cl (pH 7.5) and 150 mM NaCl]. Membranes were incubated with primary antibodies in blocking solution overnight at 4°C, washed three times for 10 min each with TBS with 0.05% Tween-20, and incubated with HRP-conjugated secondary antibodies for 1 h at room temperature in blocking solution. Membranes were washed three times for 10 min each with TBS with 0.05% Tween-20 before developing with ECL reagent. Antibodies for Mmp9 (C-20) and Gapdh (0411) were from Santa Cruz Biotechnology.

### *In situ* hybridization

Probes were synthetized from PCR fragments of mouse *Mmp9*, *Fosl1*, *Klf5* and *Creb3l1* cDNAs using primers containing promoters for T7 and Sp6 polymerases in the antisense and sense orientation, respectively. PCR fragments were obtained using cDNA clones from Open Biosystems as template. Antisense probes were obtained using T7 polymerase. Primers are listed in supplementary material Table S3. Probe hybridization was performed as described previously (Delgado-Olguin et al., 2012).

### Luciferase

Luciferase experiments were performed in bovine aortic endothelial cells (BAEC) as previously described (Wythe et al., 2013).

### Chromatin immunoprecipitation

Creb3l1- and Pol II-associated chromatin was obtained as previously described (Wythe et al., 2013) using antibodies from Abcam (Creb3l1 AB33051) and Sigma (Pol II R1530). qPCR was used to calculate the relative enrichment of Creb3l1 and RNA Pol II at the *MMP9* promoter compared with IgG control (Sigma M8695).

## Supplementary Material

Supplementary Material
