# Supplementary material for: Ezh2-mediated repression of a transcriptional pathway upstream of Mmp9 maintains integrity of the developing vasculature
Source: Development. 2014 Dec;141(23):4610–7. doi: 10.1242/dev.112607 (PMC4302930; doi:10.1242/dev.112607)
Supplement: Supplementary Material [file supp_141_23_4610__index.html]

Supplementary Material 

# Ezh2-mediated repression of a transcriptional pathway upstream of *Mmp9* maintains integrity of the developing vasculature

## DEV112607 Supplementary Material

**Files in this Data Supplement:**

- Supplementary Material
