# Supplementary material for: Ezh2-mediated repression of a transcriptional pathway upstream of Mmp9 maintains integrity of the developing vasculature
Source: Development. 2014 Dec;141(23):4610–7. doi: 10.1242/dev.112607 (PMC4302930; doi:10.1242/dev.112607)
Supplement: Supplementary Material [file supp_dev.112607_DEV112607supp.pdf]

SUPPLEMENTARY MATERIAL

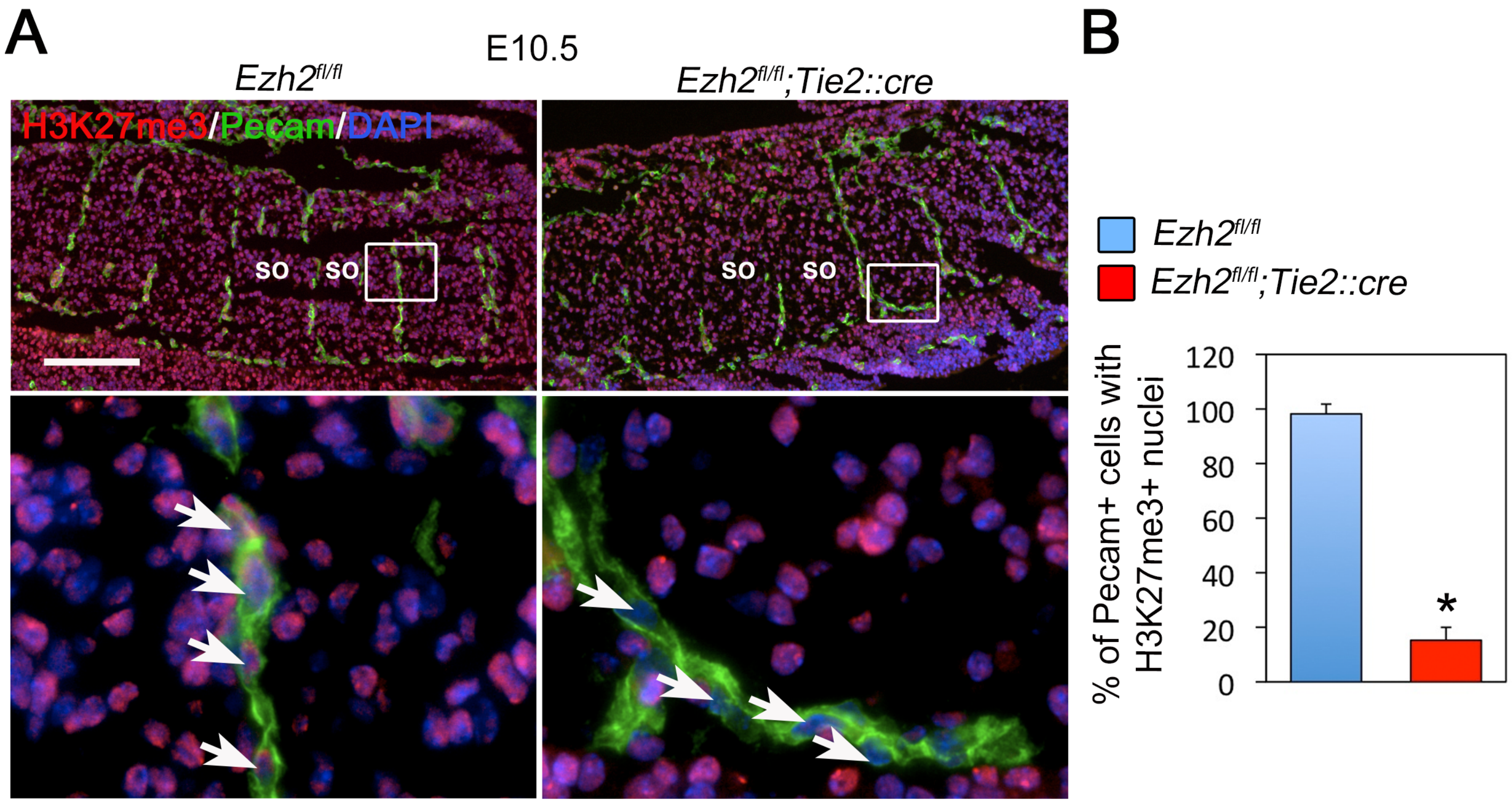

Fig. S1. Efficient Inactivation of Ezh2 in Endothelial Cells. (A) Immunofluorescence of H3K27me3 on sections of control (*Ezh2$^{fl/fl}$*) and mutant (*Ezh2$^{fl/fl}$;Tie2::cre*) mouse embryos at E10.5. Endothelial cells (green) were identified by Pecam expression. Nuclei were stained with DAPI. In contrast with controls, endothelial cell nuclei of the intersomitic vessels (arrows) of mutant embryos had decreased or absent H3K27me3. so = somite. (B) Percentage of control and mutant endothelial cell nuclei positive for H3K27me3. Bars represent the S.D of at least 100 nuclei counted in three embryos per genotype. * p<= 0.05. Scale bar = 200 μm.

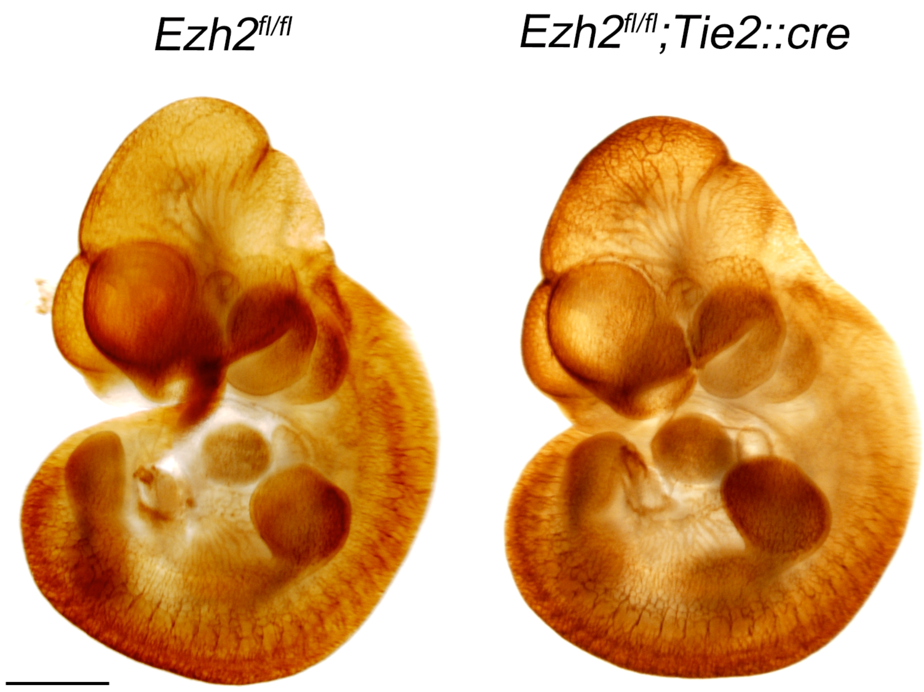

Fig. S2. Ezh2 deficiency does not affect vasculature pattern development. Whole control (*Ezh2*$^{fl/fl}$) and mutant (*Ezh2*$^{fl/fl}$*;Tie2::cre*) embryos at E10.5 stained for Pecam by immuno histochemistry. Pecam signal reveales the vasculature network by the brown precipitate. The vasculature pattern is comparable between genotypes. Scale bar 1 mm

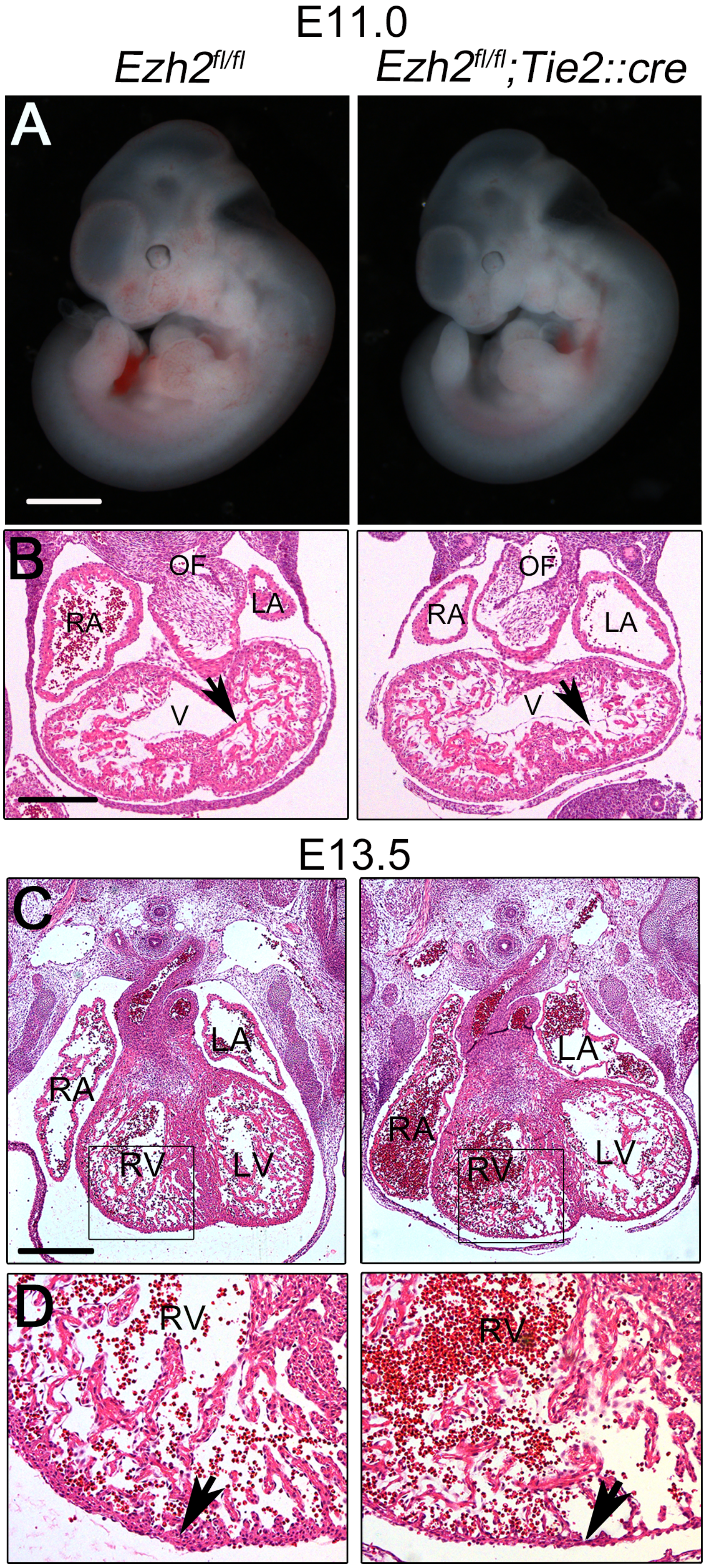

Fig. S3. Phenotype of Embryos with Ezh2-Deficient Endothelium. (A) Control (*Ezh2*$^{fl/fl}$) and mutant (*Ezh2*$^{fl/fl}$*;Tie2::cre*) embryos at E11.0. Scale bar = 1 mm. (B) Sections of control and mutant hearts showing a gap (arrow) between endocardium and myocardium. OF = outflow tract, RA = right atrium, LA = left atrium, V = ventricle. Scale bar = 200 μm. (C) Sections of control and mutant hearts at E13.5. Scale bar = 200 μm. (D) Close ups of boxes in (C). Arrow shows a thinner ventricular wall in mutant hearts. RV = right ventricle, LV = left ventricle.

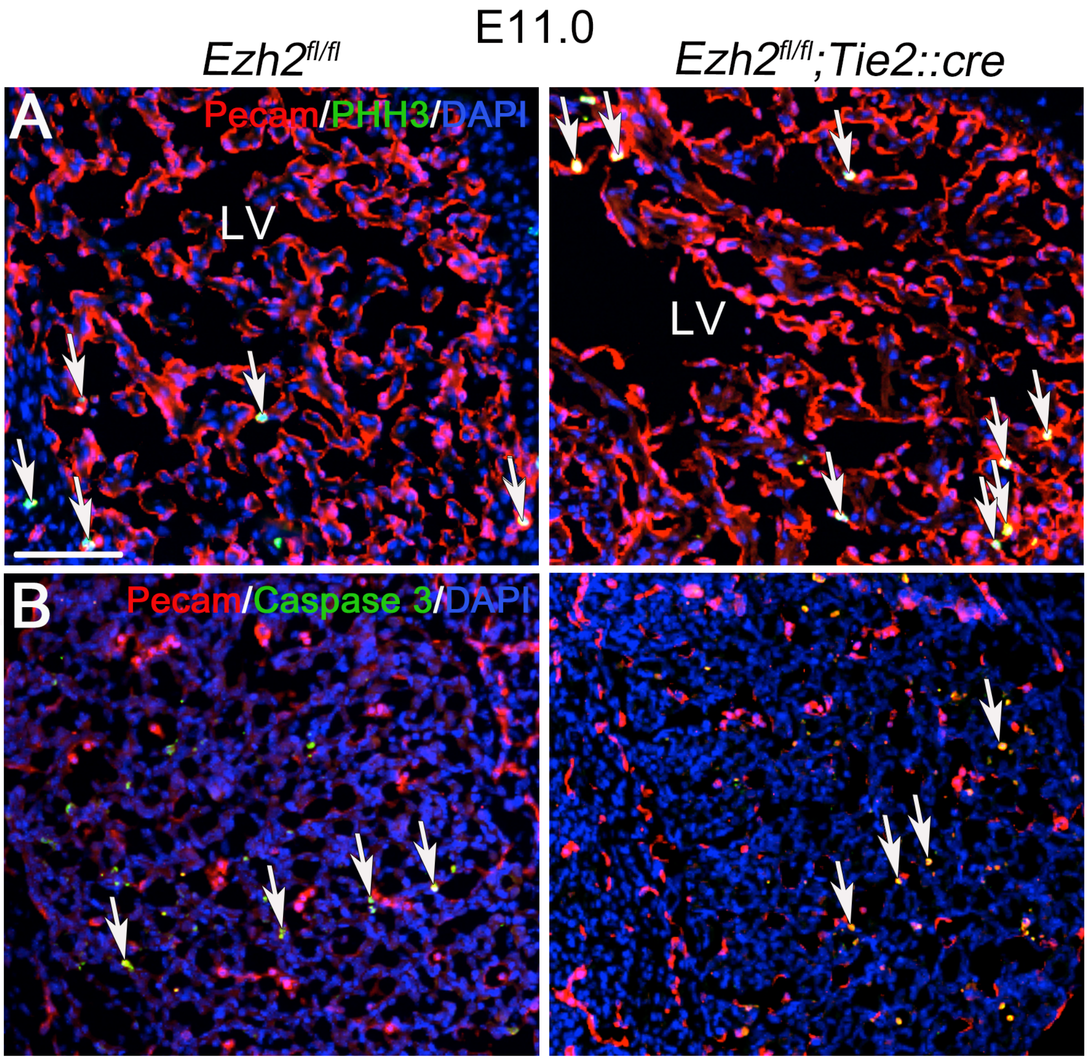

Fig. S4. Ezh2 deficiency does not affect cell proliferation or induces apoptosis. (A) Immunofluorescence of Pecam and phosphorylated histone H3 (PHH3) in sections of control (*Ezh2$^{fl/fl}$*) and mutant (*Ezh2$^{fl/fl}$;Tie2::cre*) hearts at E11.0. Nuclei were stained with DAPI. Phospho histone H3 (PHH3) positive endothelial cells (arrows) were seldom detected in control or mutant embryos, except in endocardium, where no significant differences were found between genotypes. LV = left ventricle. (B) Activated caspase 3 positive cells were seldom found in control or mutant embryos, but were found in head mesenchyme with no difference between genotypes. Scale bar = 100 μm.

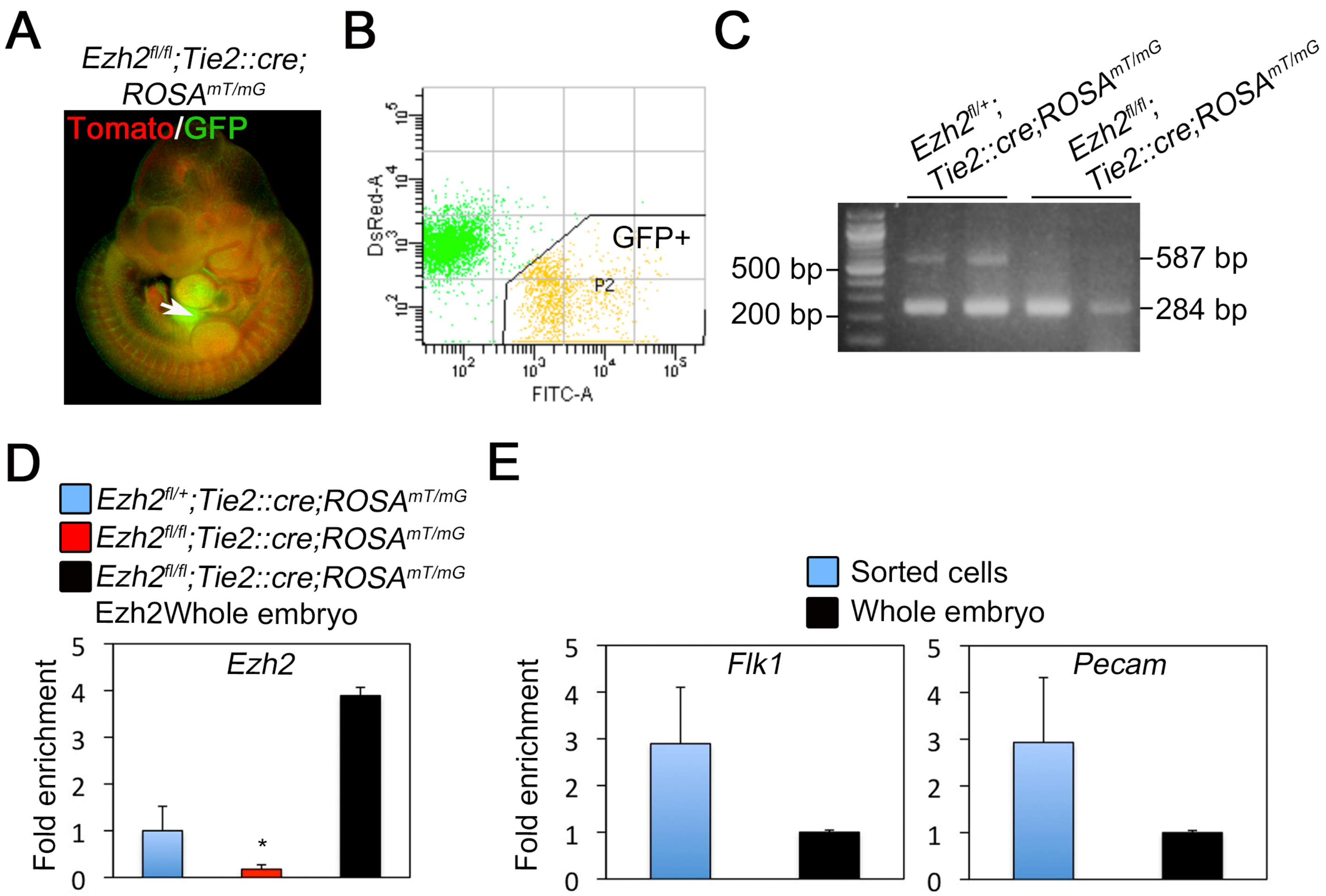

Fig. S5. Sorting of developing Ezh2-deficient endothelial cells. (A)Transgenic control embryo at E10.5 revealing *Tie2::cre* activity by expression of GFP in liver and vasculature, and tomato fluorescent protein in non-endothelial cells. (B) Plot of sorting of a GFP positive and tomato negative cell population (P2) from cre reporter embryos at E10.5. (C)Genotyping of GFP positive cells, sorted from control (*Ezh2*$^{fl/+}$*;Tie2::cre;ROSA*$^{mT/mG}$) and mutant (*Ezh2*$^{fl/fl}$*;Tie2::cre;ROSA*$^{mT/mG}$) embryos, by RT-PCR. The cDNA fragment corresponding to the wild type mRNA (587 bp) was only detected in control cells. (D) qPCR showing decreased expression of *Ezh2* in mutant vs control sorted GFP positive cells, and in endothelial cells *vs* whole embryos. (E)qPCR showing enrichment of endothelial markers *Flk1* and *Pecam* in sorted GFP positive cells. Bars represent the S.D of at least three biological replicates. * $P <= 0.05$.

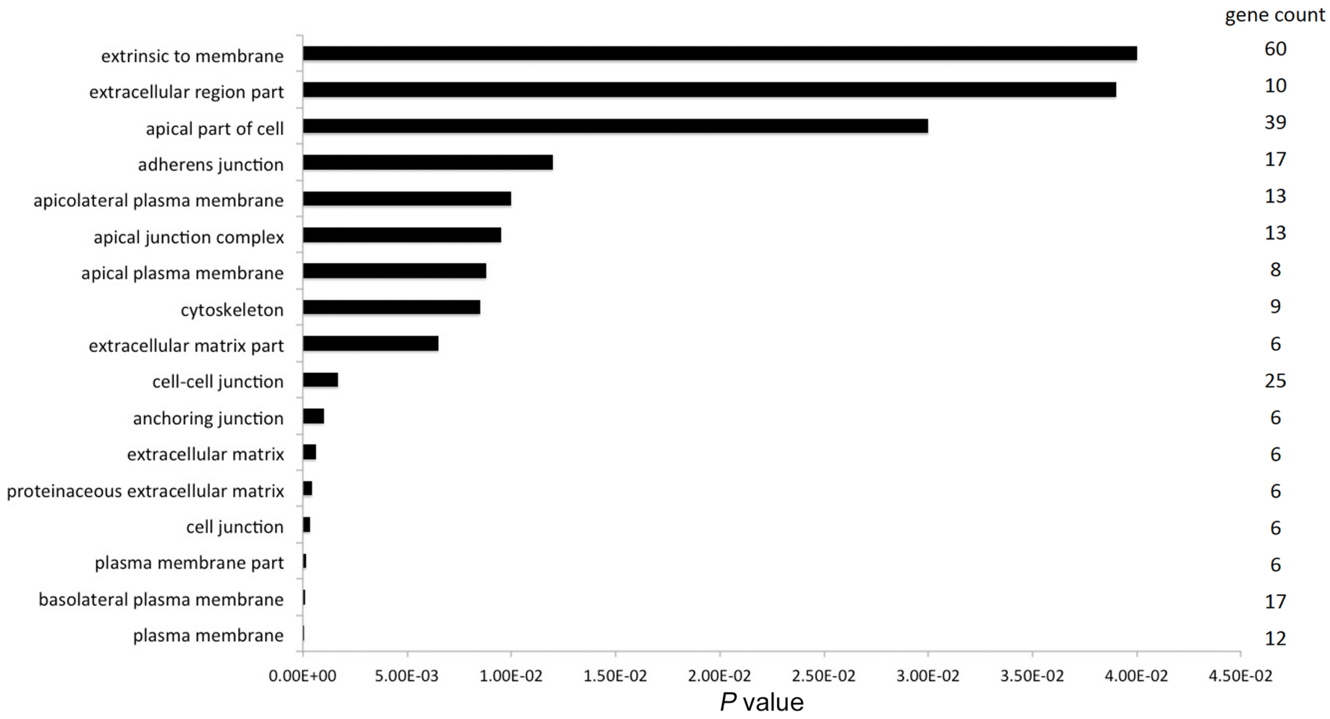

Fig. S6. Genes regulated by Ezh2 in developing endothelial cells are enriched for gene ontology categories related to cell adhesion and extracellular matrix remodeling. Genes misregulated in Ezh2-deficient endothelial cells were analyzed using the DAVID Functional Annotation Tool. Gene Ontology terms with P values < 0.05 are shown.

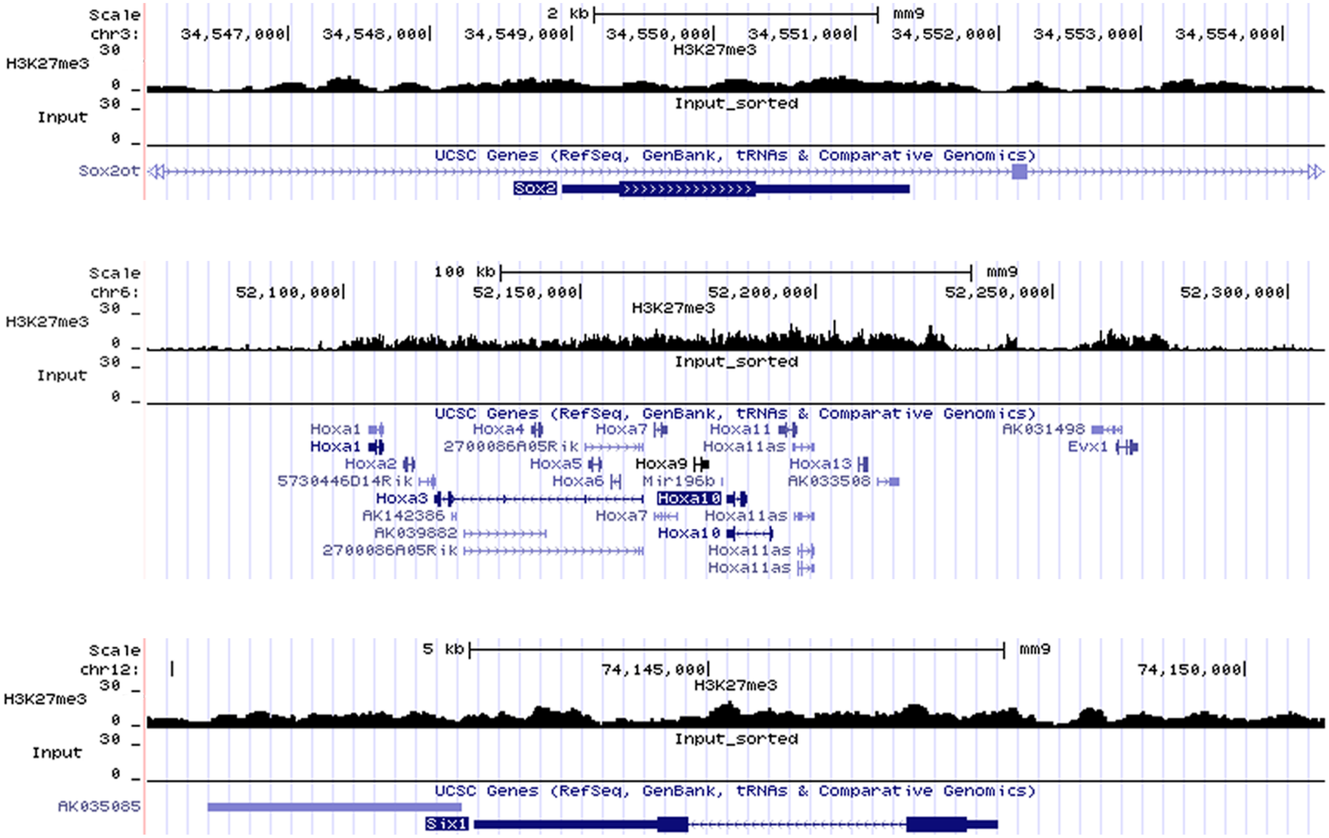

Fig. S7. Ezh2 targets *Sox2, HoxA* genes and *Six1*. USSC Genome Browser tracks of H3K27me3 immunoprecipitated from endothelial cells. H3K27me3 was enriched in previously identifed Ezh2 targets including the pluripotency factor *Sox2*, the *HoxA* gene cluster, and the homeodomain transcription factor *Six1*.

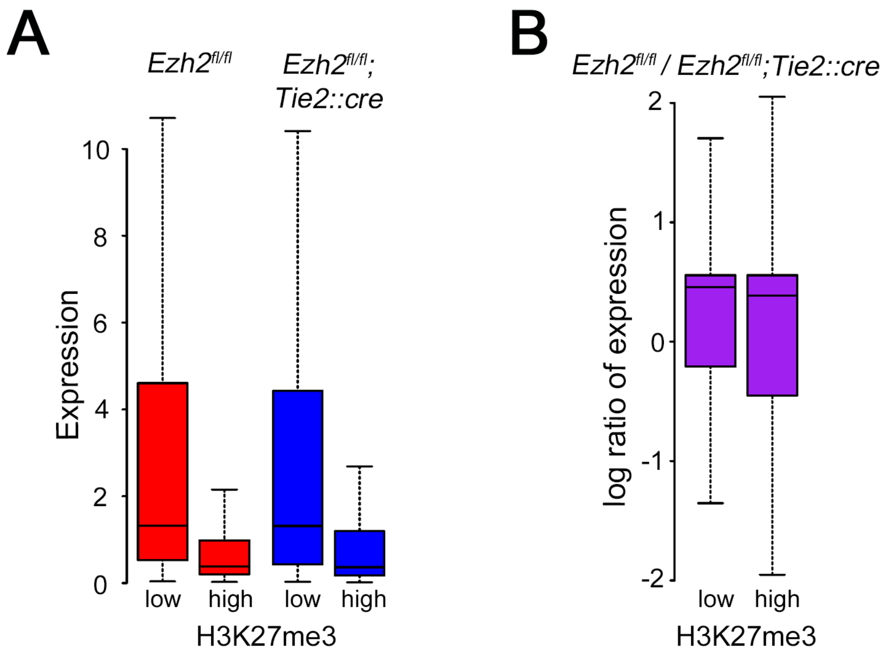

Fig. S8. H3K27me3 marked genes are not generaly predisposed towards differential expression in Ezh2-deficient endothelial cells. (A) Expression, represented as the interquartile range of scores of genes with low and high H3K27me3 enrichment for both the control (*Ezh2*$^{fl/fl}$) and mutant (*Ezh2*$^{fl/fl}$;*Tie2::cre*) datasets. (B) Interquartile range of log ratios comparing the expression of knockout to control datasets. Genes with high levels of H3K27me3 express less, while there was no overall difference between control and knockout datasets.

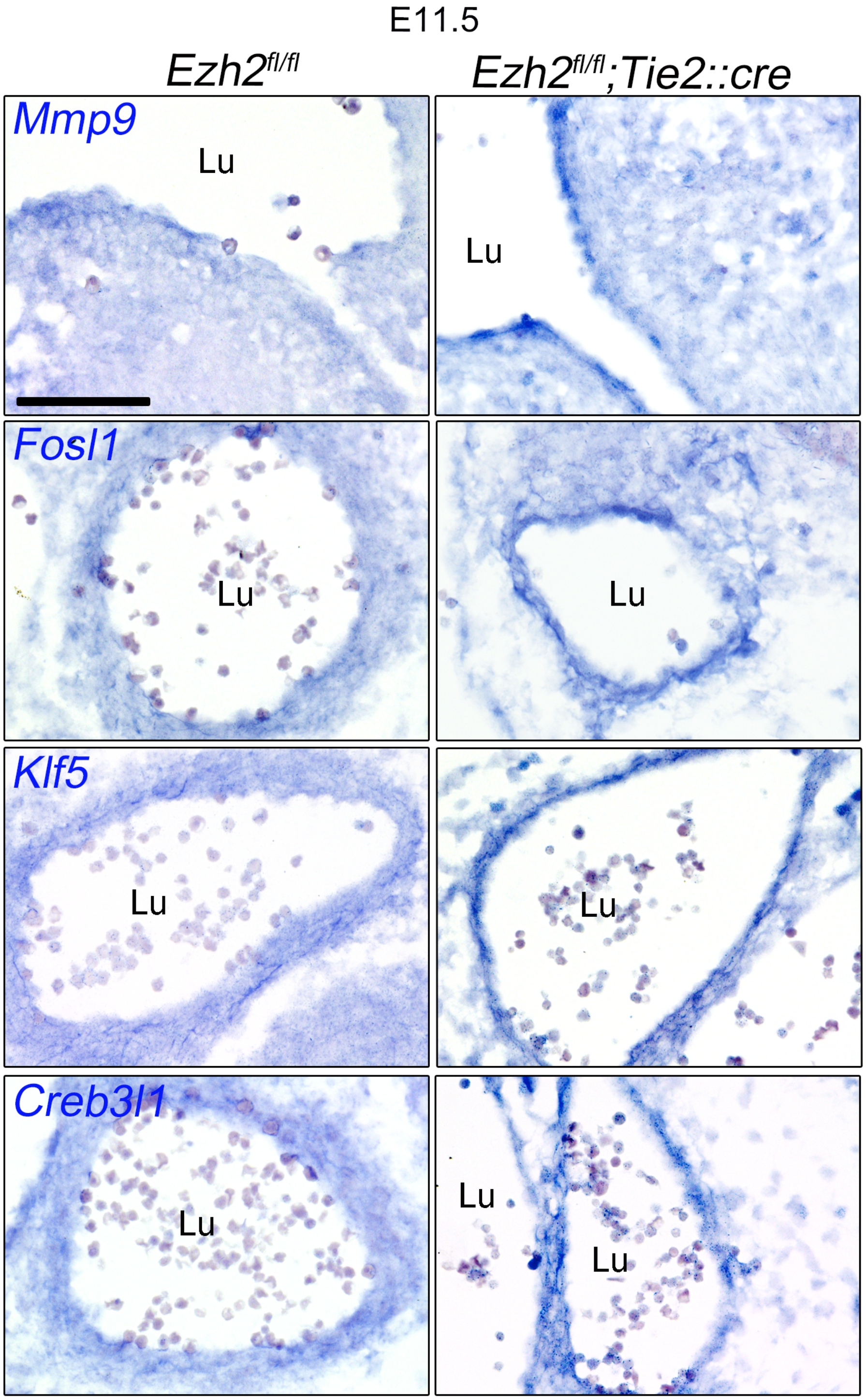

Fig. S9. *Mmp9, Fosl1, Klf5* and *Creb3l1* are upregulated in Ezh2-deficient endothelium. I*n situ* hybridization in sections of control (*Ezh2*$^{fl/fl}$) and Ezh2 mutant (*Ezh2*$^{fl/fl}$*;Tie2::cre*) embryos at E11.5. Scale bar = 50 mμ. Lu = lumen.

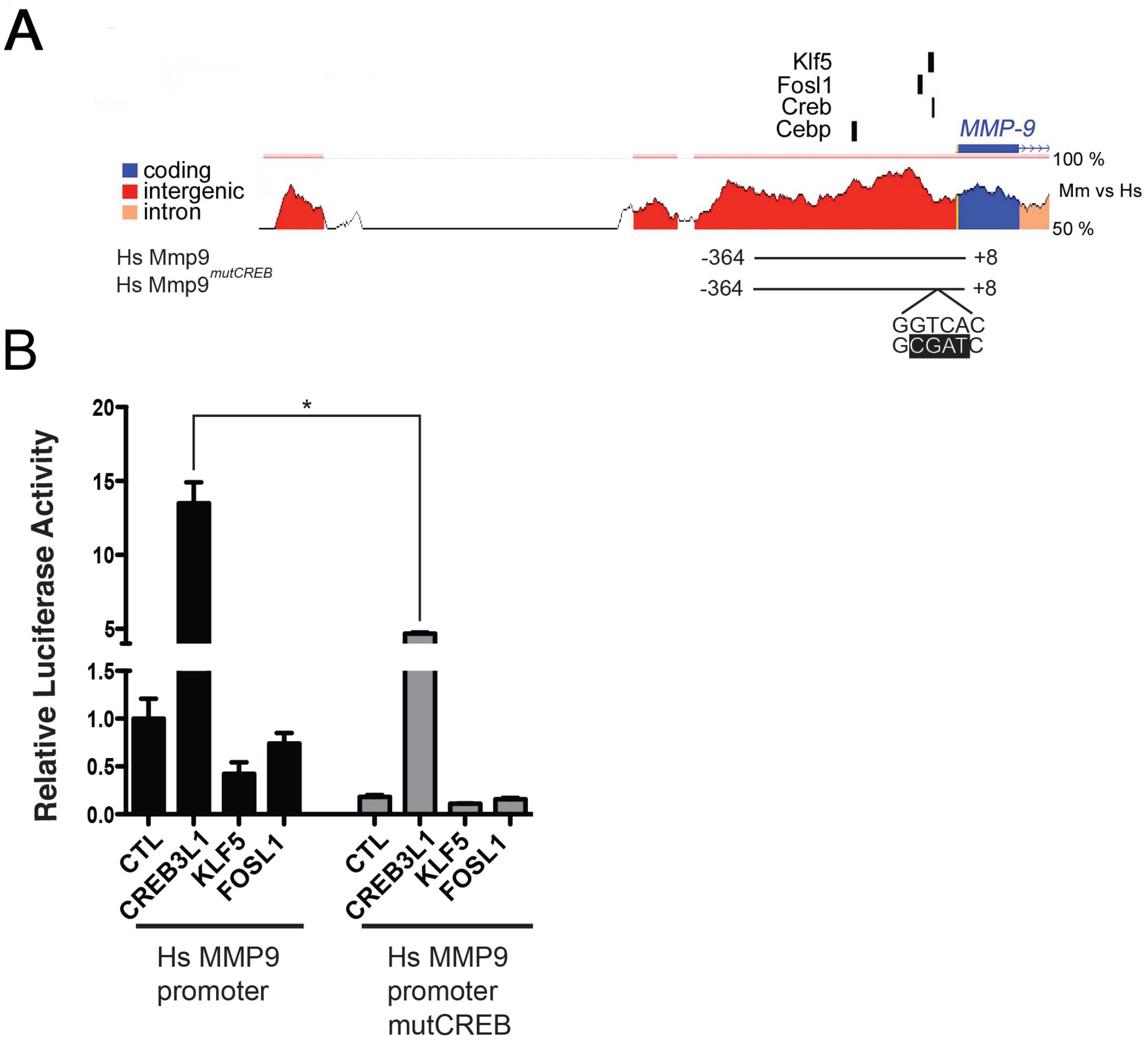

Fig. S10. CREB3L1 activates the human MMP-9 proximal promoter. (A) ECR browser conservation plots for the 5' end of the MMP-9 promoter, showing percentage conservation between mouse and human. Conserved KLF5, FOSL1, CREB3L1, and CEBPE binding motifs at the proximal promoter are indicated by black vertical lines. Line beneath plots corresponds to the human MMP-9 proximal promoter fragment used in luciferase reporter assay. CREB binding site mutation is indicated in MMP-9mutCREB. (B) Overexpression of CREB3L1, unlike FOSL1 or KLF5, can induce the human MMP-9 proximal promoter in a luciferase reporter assay. Mutation of the CREB binding site (as indicated in A) significantly reduces human MMP-9 proximal promoter activity in the presence of CREB3L1. Relative luciferase assay shown is an average of triplicates of one representative experiment.

SUPPLEMENTARY TABLES

Table S1

Click here to Download Table S1

Table S2

Click here to Download Table S2

Table S3

Click here to Download Table S3

Table S4

Click here to Download Table S4
